# Medical History from the Earliest Times

**Published:** 1892-05-07

**Authors:** E. T. Withington


### Medical History from the Earliest Times.

By E. T. Withington, M.A., M.B. Oxon.

### VII.—CHALDEAN AND PERSIAN MEDICINE.

In the opinion of Herodotus, the ancient Babylonians showed great wisdom in their treatment of the sick, for they had no physicians, but if any one was ill he was put out in the public square, and etiquette demanded that every passer-by should ask him to describe his symptoms. If the stranger had heard of a similar case, or had himself had the disease, he was expected to give advice as to treatment.

Modern discoveries confirm these statements in so far that it is very difficult to find a class of Chaldean physicians distinct from astrologers and soothsayers, and Professor Sayce tells us that the same word was used to express "physician," "scribe," and "seer." But the state of medicine revealed by the cuneiform texts by no means justifies the good opinion of the historian, for it is probably the lowest form that ever existed in a civilised community. The Chaldeans appear to have contributed absolutely nothing to the general stock of medical knowledge, and we shall, therefore, notice them very briefly. Primitive theories predominate throughout: there are the usual demons causing disease, and the usual invocations against them, both of which the reader will find fully discussed in Lenormant's "Chaldean Magic"; but Professor Sayce has recently translated fragments of a Babylonian work on medicine, which appears to indicate some progress towards a more rational practice. In it the patient sometimes has his choice whether he will use charms or medicines, and the prescriptions given comprise a considerable variety of drugs, though their precise nature cannot often be determined. It is also interesting to learn that the Babylonians were forbidden to use medicines on the sacred seventh day.

While, however, taking this unfavourable view of Chaldean medicine, it is well to remember that much yet remains to be discovered, and that the mounds of Mesopotamia may possibly still contain medical writings worthy to be compared with those of ancient Egypt.

The medicine of the Medes and Persians somewhat resembled the Chaldean, but had a still closer analogy to that of their near relatives, the Hindoos, with the important difference that it produced no Susruta or Charaka. In place of the Vedas we have the Zend-Avesta, a work ascribed to Zoroaster, but of very doubtful date and authorship. It is said to have consisted of 21 books, containing no less than 2,000,000 verses, and to have been written upon 1,200 cowhides. The healing art seems to have been very frequently mentioned; for Pliny, who had seen a Greek abstract of the entire work, declares that the religion of the Persians was evidently founded on medicine. Little of the Avesta has survived except the nineteenth book, the Vendidâd, or code of purifications—literally, "The law against demons." Here we find the famous dualistic doctrine of the government of the world by a good and an evil deity, whom, until Orientalists are more agreed in their orthography, we may still venture to call Ormazd and Ahriman.

Ahriman, according to the Vendidâd, created by his evil eye 99,999 diseases, apparently in the form of demons, whereupon Ormazd appealed for aid to Aryaman, "the friend," a god of heavenly light, who is mentioned in the Vedas. Aryaman destroys diseases by reciting the Holy Word; but Ormazd also took the 10,000 healing herbs which grew around the tree of everlasting life, and brought them to Thrita, an ancient sage and sacrificer (another Vedic personage), to whom also Kshathra-Vairya Lord of Metals, gave a knife, of which the point and base were set in gold. Thrita thus became the Persian Æsculapius, and physicians are urged to follow in his footsteps, and, like him, fight valiantly against the demons of impurity and disease. This story indicates the triple division of Persian medicine. "When physicians compete, O pure Zoroaster," says Ormazd, in another passage, "knife-doctors, herb-doctors, and word-doctors, then shall the believer go to him who heals by the holy Word, for he is a healer of healers, and benefits the soul also."

If a Persian wished to practise medicine, he must first experiment upon unbelievers; should three of these die under his hands he is for ever incapable; should he cure three, he is qualified to act as physician to the worshippers of Ormazd "for ever and ever," says the Vendidâd, though some learned commentators held that the qualification might be lost.

The Vendidâd also fixes the amount of medical fees. A priest must be healed for his blessing; the head of a house, a village, or a town for the price of an ox, of low, average, or high value respectively, while the lord of a province must pay the price of a chariot and four. The physician is also to treat animals, especially the dog, for which he must receive the value of the animal next in rank, and in the case of a sheep, the lowest on the list, his payment is the price of a "good meal."

One would have thought that a land where fees were fixed on so liberal a scale, and secured by the sanctions of religion, would have been a very paradise of physicians. But this was far from being the case. The medicine of the Medes and Persians seems to have been as conservative as their laws. The "Word-doctor" long maintained his baleful pre-eminence, and the art of healing is probably as little indebted to the land of Iran as it is to the valleys of the Euphrates and the Tigris. The Persian kings, wisely enough, entrusted themselves neither to the Chaldeans nor to their own countrymen, but got their physicians first from Egypt and afterwards from Greece; and so valued were the latter that if a practitioner in the Asiatic colonies became at all distinguished for his skill he was liable to be kidnapped and carried off to the Persian court.

In a later age, shortly before the faith of Zoroaster was driven from its ancient home by the sword of Islam, we shall find Persia the seat of an important development of medical science, but this also was mainly due to the labours, not of native, but of foreign physicians.

